# Analysis of factors associated with the prognosis of papillary thyroid cancer and the construction of a survival model

**DOI:** 10.1002/cam4.5555

**Published:** 2022-12-22

**Authors:** Peng Guo, Xinhua Wang, Luhua Xia, Nadiremu Shawureding, Zhiheng Hu

**Affiliations:** ^1^ Department of Nuclear Medicine The Affiliated Cancer Hospital of Xinjiang Medical University People's Republic of China

**Keywords:** lymph node ratio, papillary thyroid carcinoma, prognosis, thyroglobulin

## Abstract

**Objective:**

To study the survival prediction value of lymph node ratio (LNR) and preoperative thyroglobulin (Tg) in the prognosis of thyroid papillary carcinoma (PTC).

**Methods:**

A total of 495 patients with PTC and lymph node metastasis treated at the Cancer Hospital of Xinjiang Medical University were selected for a retrospective study. The disease‐free survival (DFS) of patients was the follow‐up endpoint. DFS was calculated for all patients. The Cox proportional risk regression model and nomogram were used to predict the survival prognosis of PTC with lymph node metastasis by index. LNR and preoperative Tg level cutoff values were obtained using ROC curves. To express DFS, Kaplan–Meier survival curves were created. Using 3‐ and 5‐year calibration curves and AUC values, the prognostic models' precision and discrimination were assessed. Clinical decision curve analysis was used to forecast clinical benefitability. Finally, the results were validated using internal cross‐validation.

**Results:**

The cutoff values of LNR and preoperative Tg level were 0.295 and 50.24, respectively, and they were divided into two groups according to the cutoff values. Multifactorial Cox regression models showed that NLNM, LNR, and preoperative Tg level (all *p* < 0.05) were independent risk factors affecting the prognosis of PTC with lymph node metastasis. Kaplan–Meier curves showed higher DFS rates in the group with low NLNM (<10), LNR (<0.295), and preoperative Tg level (<50.24) groups. The 3‐year and 5‐year calibration curves showed good agreement. A ROC curve analysis was performed on the nomogram model, and its AUC values at 3 and 5 years were, respectively, 0.805 and 0.793. Clinical decision curves indicate good clinical benefit. Finally, internal cross‐validation demonstrated the legitimacy of the prognostic model.

**Conclusion:**

The LNR and preoperative Tg levels, in combination with other independent factors, were effective in predicting the survival prognosis for patients with PTC.

## Graphical abstract

The value of lymph node ratio and preoperative thyroglobulin levels in predicting disease free survival in papillary thyroid cancer.

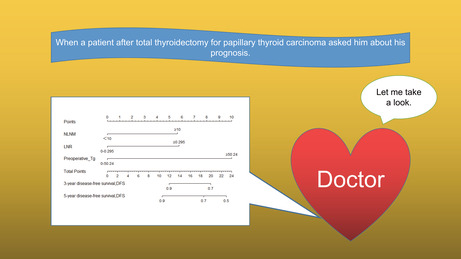

## 1 BACKGROUND

The worldwide incidence of thyroid cancer is 586,000 cases, ranking it 9th in incidence in 2020. The global incidence is three times higher in women than in men.^1^ The cause of thyroid cancer is not well understood. The only established risk factor for thyroid cancer is the effects of ionizing radiation, particularly during childhood exposure. Moreover, evidence supports that being overweight, being taller, hormone exposure (for example, thyroid stimulating hormone, thyroglobulin, etc.^2^), and certain environmental pollutants may play a role in this disease. The most prevalent form of thyroid cancer is papillary thyroid cancer (PTC),^3^, ^4^ which is often associated with lymph node metastases. Despite better overall patient survival,^5^ the recurrence rate after initial surgical treatment is high. Therefore, relapse prediction can help to clinically guide management decisions not only for patient monitoring programs but also to decide the need for further adjuvant radioiodine therapy. This prediction is particularly crucial for patients with lymph node metastases.

In patients with PTC, regional metastases in the lymph nodes play an important role in predicting tumor recurrence. The lymph node ratio (LNR), which is calculated, is the number of positive lymph nodes removed from the total number of lymph nodes removed. Several studies have shown that the LNR improves the accuracy of recurrence prediction and has also been shown to be a reliable predictor of survival and recurrence in head and neck, pancreatic, gastric, biliary, breast, colorectal, and uterine tumor types.^6^, ^7^, ^8^, ^9^, ^10^, ^11^, ^12^, ^13^, ^14^, ^15^, ^16^, ^17^, ^18^, ^19^, ^20^

Thyroglobulin (Tg) is a large glycoprotein secreted by thyroid follicular epithelial cells, most of which is synthesized by thyroid cells and released into the residual lumen of the thyroid follicle. It is a tumor marker for PTC and an important reference for the follow‐up of patients with PTC after treatment. In recent years, more studies have shown the role of preoperative Tg in lymph node metastasis and distant metastasis^21^, ^22^, ^23^, ^24^ and the preoperative identification of follicular thyroid cancer and radioiodine refractory treatment.^25^, ^26^ However, fewer studies have shown the prognostic relevance of preoperative Tg to patients.

The previous study used only LNR alone to determine the prognostic relationship with PTC; the present study evaluated performed the combination of preoperative Tg and LNR to predict the prognosis of patients. Therefore, this study attempts to construct a predictive nomogram with the LNR and preoperative serum Tg level to improve the accuracy of treatment and follow‐up plans for patients with lymph node metastases of PTC, providing a valid reference tool.

## 2 MATERIALS AND METHODS

From January 2010 to December 2020, 495 patients with pathologically confirmed PTC who underwent radical surgery at the Affiliated Cancer Hospital of Xinjiang Medical University were selected for a retrospective study. All patients were staged pathologically according to the 8th edition of TNM staging jointly developed by the American Joint Committee on Cancer (AJCC) and the Union for International Cancer Control (UICC). Inclusion criteria are: (1) PTC with histopathologically confirmed lymph node metastases from January 2010 to December 2020; (2) no preoperative chemotherapy and/or radiotherapy; and (3) serum Tg was collected in the morning 3 days before surgery. Exclusion criteria: (1) previous or concurrent other malignancies; or (2) missing clinical information and data. The last follow‐up date was December 31, 2021. Recurrence was identified based on the following criteria: (1) serologically suppressive Tg ≥1 μg/L or irritant Tg ≥10 μg/L or an increasing trend in Tg antibody (TgAb); (2) imaging verifiable evidence of the presence of structural or functional disease; and (3) recurrence followed by surgery and histopathological confirmation.

### 2.1 Statistical analysis

Optimal cutoff values for continuous variables were obtained from subject characteristic curves (ROCs). Rates were compared between groups using the chi‐square test. The Spearman correlation coefficient indicated the correlation between study indicators and pathological parameters. Cox regression analysis models were used to perform one‐way and multifactorial analyses to obtain independent risk factors affecting survival; Cox regression coefficients were used to generate nomograms. Kaplan–Meier survival curves were made to pinpoint disease‐free survival (DFS). Accuracy and non‐specificity were expressed using calibration curves and AUC values; clinical benefit was expressed by clinical decision curve analysis; and finally, internal validation was performed. All analyses with *p* < 0.05 were statistically significant. SPSS 26.0 software and R (foreign), (survival), and (rms) software packages were used for statistical analysis.

## 3 RESULTS

### 3.1 Cutoff values for continuous variables of study indicators

**FIGURE 1:**
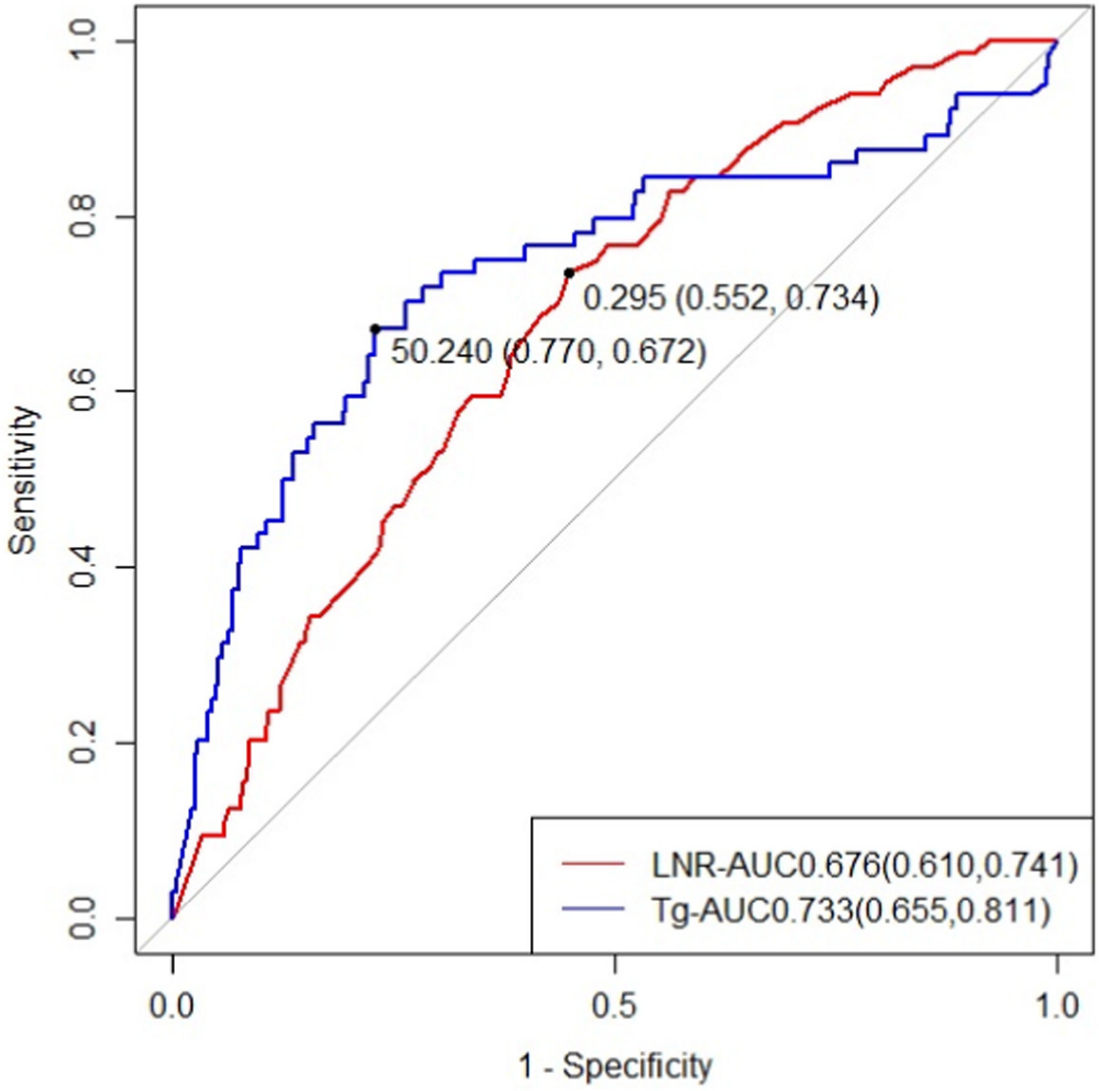
ROC curves of LNR and preoperative Tg levels. LNR: lymph node ratio; Tg: thyroglobulin.

The ROC curve was used to find out whether the LNR and preoperative Tg levels had appropriate thresholds to predict the patient's final outcome of recurrence or death, which suggested that these indicators may be significant for prognosis. The best critical value for predicting DFS for LNR was 0.295, which was associated with the best sensitivity (0.734) and specificity (0.552). The best critical value for predicting preoperative Tg levels for DFS was 50.24, which was associated with the best sensitivity (0.672) and specificity (0.770) (Figure 1).

### 3.2 Correlation of LNR and preoperative Tg levels with clinicopathological features

Among the clinicopathological characteristics of the selected patients (Table 1), the number of lymph node metastases (NLNM) was grouped based on the ROC cutoff value to determine. The female to male ratio was approximately 2.41:1. The proportion of patients aged <55 years (86.5%) was significantly higher than that of patients aged ≥55 years (13.5%). The ratio of patients without Hashimoto's thyroiditis (HT) to those with HT was approximately 2.78:1. Stage I (86.5%) and stage II (10.9%) predominated in pathological staging. The proportion of lymph node metastasis N1b (56.2%) was higher than that of stage N1a (43.8%). The majority of tumors had a maximum diameter of ≤2 cm (75.2%), which may be related to the common use of imaging examinations such as ultrasound and CT, which improve the detection of early lesions.

**TABLE 1:** Clinicopathological characteristics of PTC and each study index

Characteristic	Number of patients(*n* = 495)	Composition ratio(%)	*χ* ^2^	*p*
Sex				
Female	350	70.7%	1.567	0.211
Male	145	29.3%		
Age				
<55 years	428	86.5%	0.838	0.36
≥55 years	67	13.5%		
MTD				
≤2 cm	372	75.2%	18.172	<0.001
>2 cm, ≤4 cm	97	19.6%		
>4 cm	26	5.3%		
NTF				
Unifocal	219	44.2%	2.055	0.152
Multifocal	276	55.8%		
MDLNM				
<1.15 cm	390	78.8%	14.019	0.001
≥1.15 cm, <3 cm	101	20.4%		
≥3 cm	4	0.8%		
NLNM				
<10	382	77.2%	42.349	<0.001
≥10	113	22.8%		
LNR				
<0.295	255	51.5%	18.323	<0.001
≥0.295	240	48.5%		
Pathological_stage				
I	428	86.5%	8.079	0.044
II	54	10.9%		
III	11	2.2%		
IVA	2	0.4%		
T_stage				
T1	301	60.8%	22.1	<0.001
T2	60	12.1%		
T3	81	16.4%		
T4a	49	9.9%		
T4b	4	0.8%		
N_stage				
N1a	217	43.8%	8.91	0.003
N1b	278	56.2%		
HT				
No	364	73.5%	1.43	0.232
Yes	131	26.5%		
Preoperative_Tg				
<50.24	353	71.3%	53.259	<0.001
≥50.24	142	28.7%		

Abbreviations: HT, Hashimoto's thyroiditis; LNR, lymph node ratio; MDLNM, maximum diameter of lymph node metastasis; MTD, maximum tumor dimension; NLNM, number of lymph node metastases; NTF, number of tumor foci.

Based on the correlation between the selected study indicators and pathological parameters (Table 2), the LNR was significantly correlated with sex (*p* = 0.004) and NLNM (*p* < 0.001). Preoperative Tg levels were significantly correlated with maximum tumor dimension (MTD) (*p* < 0.001), maximum diameter of lymph node metastasis (MDLNM) (*p* = 0.002), NLNM (*p* < 0.001), HT (p < 0.001), T stage (*p* < 0.001), and N stage (*p* < 0.001).

**TABLE 2:** Correlation of pathological features of PTC with LNR and preoperative Tg levels

Variables	Cases	LNR		R	*p*	Preoperative_Tg		R	*p*
		<0.295	≥0.295			<50.240ng/ml	≥50.240		
Sex									
Female	350	195	155	0.131	0.004	251	99	0.014	0.76
Male	145	60	85			102	43		
Age									
<55 years	428	216	212	−0.053	0.239	310	118	0.062	0.166
≥55 years	67	39	28			43	24		
MTD									
≤2 cm	372	200	172	0.084	0.063	295	77	0.324	<0.001
>2 cm,≤4 cm	97	46	51			53	44		
>4 cm	26	9	17			5	21		
NTF									
Unifocal	219	110	109	−0.023	0.611	164	55	0.07	0.118
Multifocal	276	145	131			189	87		
MDLNM									
<1.15 cm	390	207	183	0.06	0.184	291	99	0.141	0.002
≥1.15 cm, <3 cm	101	46	55			60	41		
≥3 cm	4	2	2			2	2		
NLNM									
<10	382	233	149	0.349	<0.001	294	88	0.23	<0.001
≥10	113	22	91			59	54		
Pathological_stage									
I	428	216	212	−0.047	0.296	310	118	0.072	0.11
II	54	35	19			40	14		
III	11	4	7			3	8		
IVA	2	0	2			0	2		
T_stage									
T1	301	160	141	0.036	0.422	243	58	0.256	<0.001
T2	60	28	32			31	29		
T3	81	41	40			54	27		
T4a	49	24	25			24	25		
T4b	4	2	2			1	3		
N_stage									
N1a	217	116	101	0.034	0.446	174	43	0.173	<0.001
N1b	278	139	139			179	99		
HT									
No	364	178	186	−0.087	0.053	239	125	−0.208	<0.001
Yes	131	77	54			114	17		

Abbreviations: HT, Hashimoto's thyroiditis; LNR, lymph node ratio; MDLNM, maximum diameter of lymph node metastasis; MTD, maximum tumor dimension; NLNM, number of lymph node metastases; NTF, number of tumor foci.

### 3.3 One‐way and Multifactorial Cox regression analysis of each study indicator

**FIGURE 2:**
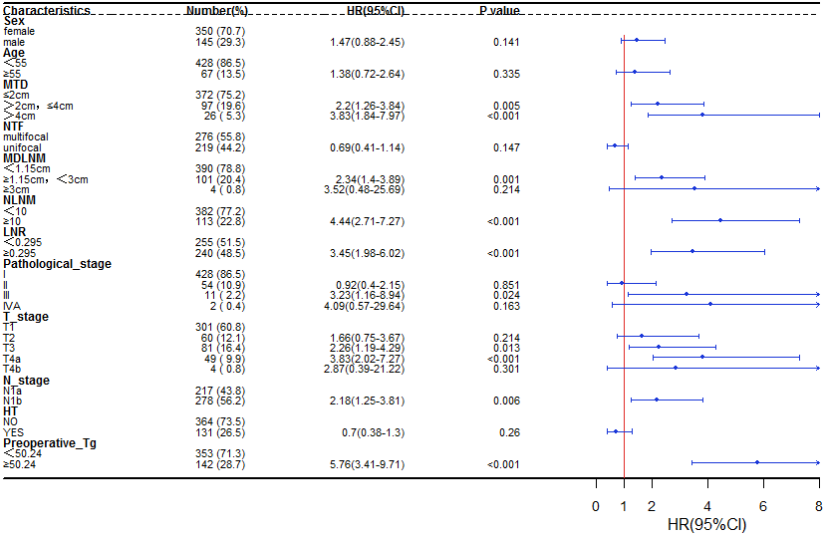
Single factor forest diagram.HT, Hashimoto's thyroiditis; LNR, lymph node ratio; MDLNM, maximum diameter of lymph node metastasis; MTD, maximum tumor dimension; NLNM, number of lymph node metastases; NTF, number of tumor foci.

One‐way Cox regression analysis showed that sex, age, MTD, number of tumor foci (NTF), MDLNM, NLM, LNR, pathological stage, T stage, N stage, HT and preoperative Tg level. MTD (*p* < 0.001), MDLNM (*p* = 0.001), NLNM (*p* < 0.001), LNR (*p* < 0.001), pathological stage (*p* = 0.024), T stage (*p* < 0.001), N stage (*p* = 0.006) and preoperative Tg level (*p* < 0.001) were prognostic factors for PTC with lymph node metastasis (Figure 2). Multifactorial Cox regression analysis revealed that NLNM (*p* = 0.002), LNR (*p* = 0.004) and preoperative Tg level (*p* < 0.001) were independent risk factors affecting the prognosis of PTC with lymph node metastasis (Table 3). The risk of recurrence was 2.395 times higher in the group with a LNR ≥0.295 than in the <0.295 group (95% CI: 1.328–4.321). The risk of recurrence was 4.554 times greater in the group with preoperative Tg levels ≥50.24 compared to the <50.24 group (95% CI: 2.667–7.777).

**TABLE 3:** Multifactor analysis

Characteristics	B	SE	Wald	*p*	HR(95%CI)
NLNM	0.856	0.271	9.952	0.002	2.354(1.383, 4.006)
LNR	0.873	0.301	8.146	0.004	2.395(1.328, 4.321)
Preoperative_Tg	1.516	0.273	30.821	<0.001	4.554(2.667, 7.777)

Abbreviations: LNR, lymph node ratio; NLNM, number of lymph node metastases.

### 3.4 Survival analysis for significance testing indicators to create survival curves

**FIGURE 3:**
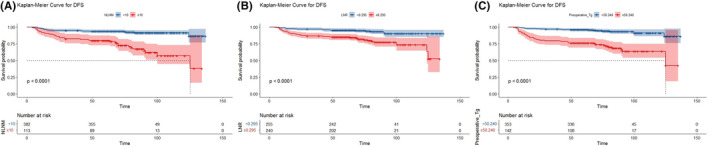
(A–C) Survival curve of NLNM, LNR, and preoperative Tg level for predicting DFS

Based on the results of Cox regression analysis, the NLNM, LNR, and preoperative Tg levels were chosen to establish the DFS survival curve. Figure 3A–C indicates a higher DFS rate in the NLNM (<10) group, LNR (<0.295) group, and preoperative Tg (<50.24) groups.

### 3.5 Construction and internal validation of a column line graph for predicting DFS

**FIGURE 4:**
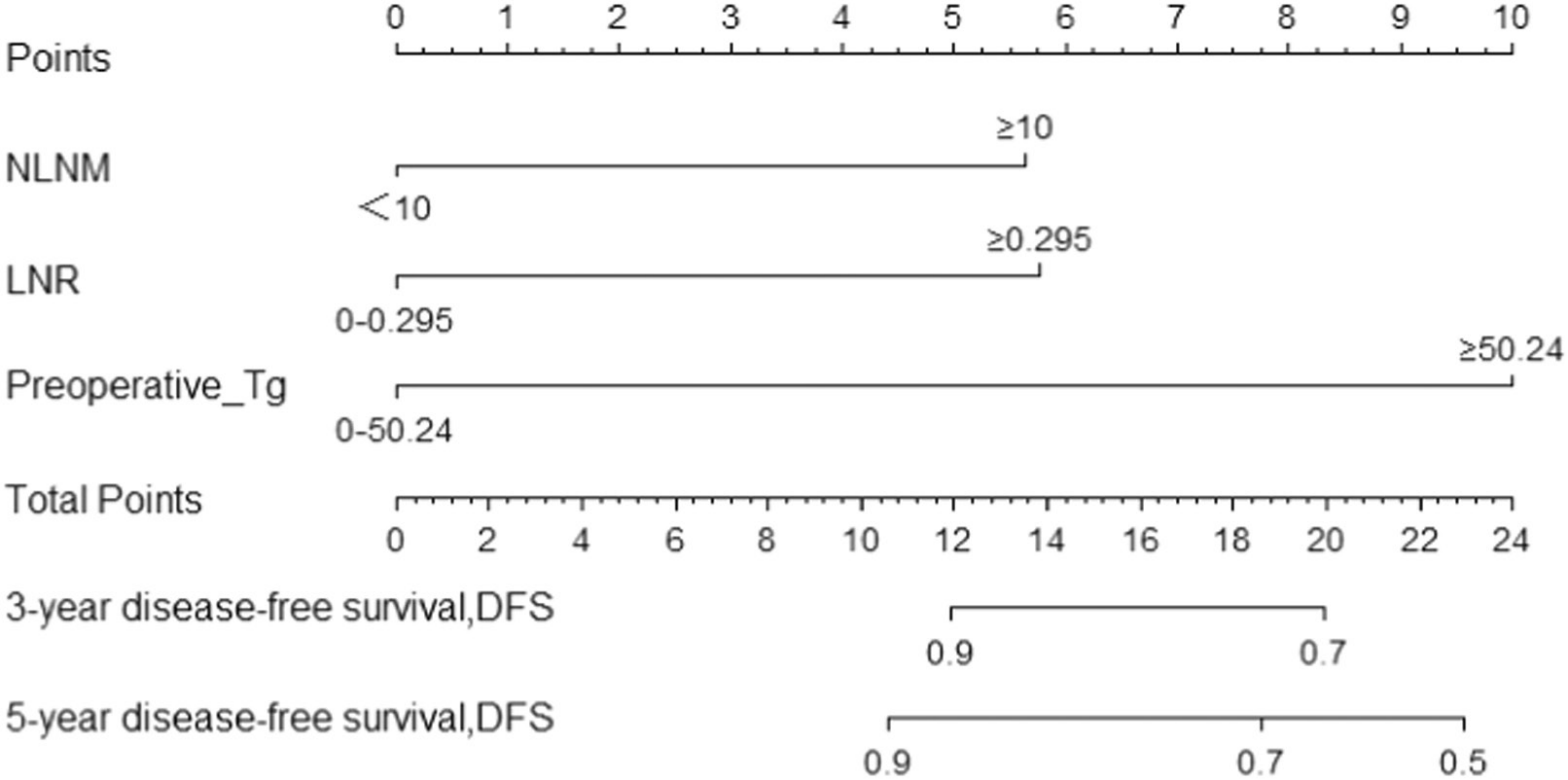
Construction of DFS nomogram to predict PTC

**FIGURE 5:**
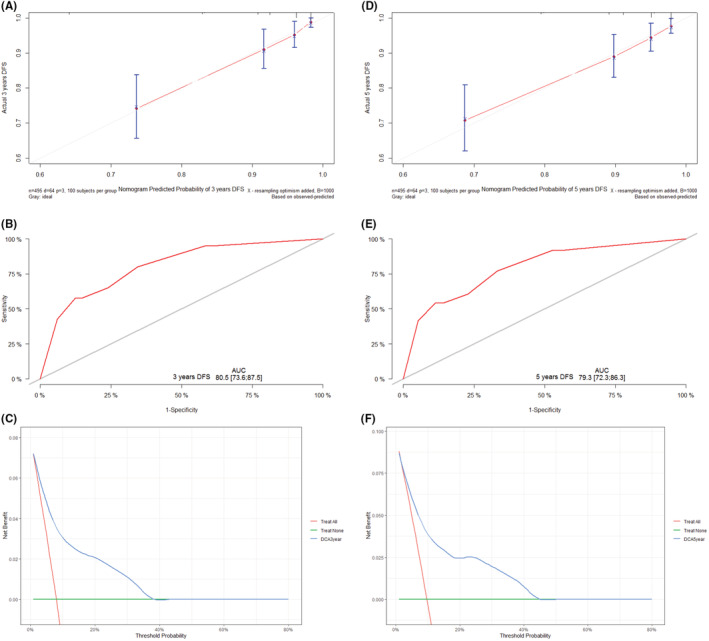
(A–C) 3‐year calibration curves, ROC curves, and clinical decision curves; (D–F) 5‐year calibration curves, ROC curves, and clinical decision curves

Cox regression‐based multifactorial analysis: Nomograms predicting 3‐ and 5‐year DFS by NLNM, LNR, and preoperative Tg levels were established (Figure 4). The calibration curves for DFS at 3 and 5 years showed good agreement between the predicted and actual values of the nomogram. The ROC curve analysis of the nomogram model showed good discriminatory ability, with AUC values of 0.805 (95% CI: 0.736–0.875) and 0.793 (95% CI: 0.723–0.863) for 3‐ and 5‐year survival, respectively. Clinical decision curves indicate good clinical benefit (Figure 5).

Subsequent internal cross‐validation of the constructed nomogram model revealed AUC values of 0.798 and 0.786 for 3 and 5 years, respectively. These results suggest that the nomogram is a good predictor of DFS in PTC with lymph node metastases.

## 4 DISCUSSION

Lymph node metastasis in PTC is now generally accepted to be strongly associated with an increased risk of recurrence, with only the number of lymph nodes classified in the latest risk stratification for recurrence. In many studies, LNR has been found to correlate with patients' risk of recurrence, and it has even been shown to be superior to traditional prognostic factors such as TNM classification, age, and sex. In clinical staging guidelines, N stage is divided into N1a and N1b based on the region of metastasis, which may understate the significance and extent of the metastatic burden of the disease. In this study, we discuss the prognostic value in terms of LNR and preoperative Tg levels.

In the present study, we found a strong correlation between the LNR, sex, and the number of migrating lymph nodes and found that the LNR (≥0.295) was an independent influencing factor for PTC. This finding is in line with most studies. In a study of PTC in children and adolescents,^27^ a high LNR (>0.34) was found to be an independent predictor of persistent disease after initial treatment, with a three‐fold higher risk of persistent disease after initial treatment compared to corresponding patients. Jill C. Rubinstein et al.^28^ found that the LNR was an important predictor for stratifying the likelihood of recurrence of PTC in children in a study of 48 patients with PTC, with patients in the high LNR group (>0.45) having an increased rate of recurrence. Moreover, recurrence rates were higher for elderly patients in the high central compartment LNR group (>0.5).^29^ Sang Hun Lee et al.^30^ found that posttreatment recurrence predicted lateral compartment and postoperative serum thyroglobulin levels in patients with lateral neck metastatic PTC (N1b) based on the patient's LNR (>0.25). Additionally, Moran Amit et al.^31^ showed the reproducibility of the LNR (>0.19) as a predictor of prognosis in PTC. Kim, H I et al.^32^ also suggested that grouping N1b patients using a risk assessment method that includes a high LNR (>0.3) can better distinguish between patients with a poor prognosis and those with a good prognosis than the eighth edition of the TNM staging system. Additionally, Parvathareddy, S K et al.^33^ also found that combining LNR staging with the 8th edition of the AJCC TNM staging system and ATA risk stratification would improve the accuracy of predicting recurrence. The differences in cutoff values may be related to the different nadir criteria of each study and the method of determining the cutoff values. LNR has been increasingly shown to have prognostic value, but more research is still needed to determine the ideal cutoff value for LNR, allowing uniformity of the extent and number of surgically removed lymph nodes.

This study found that the preoperative Tg level was closely correlated with MTD, MDLNM, NLNM, HT, T stage, and N stage. Moreover, this study found that the preoperative Tg level independently influenced the prognosis of PTC. Therefore, we should pay more attention to the preoperative high Tg level of PTC, which can provide a reference for the prognosis of patients.

Therefore, we attempted to construct a nomogram based on LNR, preoperative Tg levels, and clinicopathological parameters to predict prognosis. The calibration curves for 3‐ and 5‐year survival demonstrated the consistency of the predicted probabilities with the actual probabilities, and the clinical decision curves demonstrated the predictive potential of the nomogram. Moreover, internal validation was conducted.

Our model has several limitations. First, this study is a review, and in spite of the fact that it examined a large sample, LNR are influenced by the management of clinical surgical scopes and the number of lymph nodes recovered, which may significantly affect the outcome of LNR, and our pathologists only retrospectively analyzed the pathology reports. Second, we did not perform a study to differentiate between N1a and N1b subdivisional LNR. In addition, the number and dose of radioiodine already used postoperatively were not included in the analysis in this study. Finally, our study has only been internally validated, and multi‐institutional, multicenter, and more forward‐looking studies are needed to validate our results.

## AUTHOR CONTRIBUTIONS

**Peng Guo:** Conceptualization (lead); data curation (lead); methodology (lead); writing – original draft (equal). **Xinhua Wang:** Conceptualization (lead); formal analysis (equal); methodology (equal); writing – original draft (lead). **Luhua Xia:** Data curation (equal); methodology (equal); writing – original draft (equal). **Nadiremu Shawureding:** Data curation (equal); methodology (equal); writing – original draft (equal). **Zhiheng Hu:** Data curation (equal); methodology (equal); writing – original draft (equal).

## FUNDING INFORMATION

No funding.

## CONFLICT OF INTEREST

None declared.

## DECLARATIONS

The study was approved by Ethics Committee of the Cancer Hospital Affiliated to Xinjiang Medical University. All individuals participating in the study signed an informed consent form.

## Data Availability

I confirm that my article contains a Data Availability Statement even if no data is available (list of sample statements) unless my article type does not require one.
